# Experiencing Cultural Safety in Nursing Care: A Focused Ethnography With Refugees

**DOI:** 10.1111/nhs.70311

**Published:** 2026-02-18

**Authors:** Maki Nakajima, Ikumi Honda, Tomoko Doi

**Affiliations:** ^1^ Graduate School of Medicine, Department of Integrated Health Sciences Nagoya University Nagoya Japan; ^2^ Faculty of Nursing Nagoya University of Arts and Sciences Nagoya Japan

**Keywords:** cultural safety, health equity, Japan, nursing care, qualitative research, refugees

## Abstract

This study explored healthcare and nursing care experiences of Laotian refugees in Japan, focusing on their interpretations of cultural safety. A focused ethnography was conducted, incorporating semi‐structured interviews and participant observations with 20 Laotian refugees at temples, homes, and community events. Thematic analysis, informed by the interpretive lens of cultural safety, guided interpretation. Five major themes were identified: Having a place of spiritual sanctuary, culturally congruent care, hopes for holistic care within the Japanese healthcare system (body–mind–spirit), recognition, and equality from Japanese society, and receiving culturally respectful end‐of‐life care. Themes revealed how spiritual, cultural, and social continuity shape refugees' health experiences, highlighting both resilience and vulnerability. Nurses can foster culturally safe care by supporting spiritual and community spaces, integrating bicultural practices, addressing systemic inequities, and collaborating with families and community leaders in end‐of‐life planning.

## Key Points

- Cultural safety in nursing care fosters refugees' trust, dignity, and participation by recognizing identity and addressing power imbalances within healthcare relationships.
- Temples function as culturally safe community health resources that support refugees' emotional, spiritual, and social well‐being alongside biomedical care.
- Equity‐oriented nursing practice requires reflection on power, collaboration with communities, and contextually tailored approaches to care for culturally diverse populations.

## 1 Introduction

During the COVID‐19 pandemic, health and social inequalities experienced by refugees intensified, driven by reduced support systems and limited access to accurate health information (Nichol et al. 2022). Refugees' access to healthcare is often restricted by language barriers, cultural differences, and institutional constraints, further complicated by divergences between refugees' health beliefs and those of nurses in host countries as well as limited familiarity with host healthcare systems (International Council of Nurses 2023). Nurses play a central role in addressing these obstacles by empathizing with refugee experiences (Simich et al. 2003) and valuing their perspectives (Davenport 2017). Culturally responsive nursing care that bridges differences and respects refugees' values is essential for equitable care (Grigg‐Saito et al. 2010; Magwood et al. 2022). Additionally, when care fails to align with cultural expectations, nurses may also experience distress (Epstein et al. 2019).

In Japan, refugees face persistent barriers to healthcare access, and health disparities remain a major concern. For instance, the Japanese healthcare system lacks comprehensive and routinely available health interpreting services, with language‐related barriers and structural constraints repeatedly identified as impediments to equitable healthcare access for migrants and refugees (Khin et al. 2025). During the pandemic, the introduction of specialized healthcare services added layers of complexity, creating further obstacles for migrants and refugees with limited Japanese proficiency and health literacy (Yamashita et al. 2024). These were compounded by fear and xenophobia, amplifying feelings of exclusion and vulnerability. Thus, cultural safety has become increasingly critical, requiring not only technically competent care but also healthcare interactions that affirm identity, foster trust, and address structural inequities (So et al. 2024). For refugees, cultural safety provides a framework for understanding how respect, participation, and equality can be realized in healthcare, making it indispensable for advancing equitable nursing practice.

## 2 Background

### 2.1 Refugees in Japan and Research Gaps

Japan, a signatory of the Refugee Convention, began accepting Indochinese refugees from Vietnam, Laos, and Cambodia in 1979. Under Japan's government‐led resettlement program, a total of 11 319 Indochinese refugees were accepted, including 1306 refugees from Laos. A long‐term resident population of 25 000 Indochinese refugees and their families has thus formed (Refugee Assistance Headquarters 2024). Most research on Indochinese refugee health in Japan has focused on the early years following resettlement, and while mental health risks have been examined extensively, there is less attention on physical health and nursing perspectives (Desmyth et al. 2021). Over recent decades, refugee demographics of age and country of origin have diversified, and the need for healthcare services has increased. Yet, no refugee‐specific medical services have been established. Moreover, overseas residents who have applied for refugee status are disproportionately unlikely to seek medical treatment, reflecting structural and social barriers (Morita et al. 2021). Inadequate support systems exacerbate these challenges, underscoring the importance of understanding refugees' perspectives to inform culturally safe nursing care.

Few studies have distinguished the specific experiences of Laotian refugees; little is known about their lived experiences of healthcare and nursing care, and no previous studies have explicitly applied the lens of cultural safety. This gap is significant, as perceptions of health and illness strongly influence healthcare‐seeking behaviors, communication with providers, and trust in the care system (Kirmayer et al. 2011). Research shows that incorporating refugee patients' cultural narratives into care planning fosters trust, engagement, and improved health outcomes (Edge and Newbold 2013).

### 2.2 Study Purpose

The study aim was to explore how Laotian refugees in Japan describe and interpret their experiences of healthcare and nursing care in relation to cultural safety. The guiding research question was “How do Laotian refugees in Japan describe and interpret their experiences of healthcare and nursing care in relation to cultural safety?” Findings aim to inform culturally safe nursing practice in Japan and contribute to global and Asia‐Pacific discussions on refugee health equity.

## 3 Methods

### 3.1 Research Paradigm and Reflexivity

This study was grounded in an interpretivist paradigm, emphasizing how participants construct meanings of health, illness, and healthcare within sociocultural contexts. The first author, a Japanese nursing researcher with experience in refugee health support, had lived in Laos and speaks Lao, which helped build rapport. Reflexivity was maintained through field notes and memos to account for positionality and prior relationships, enhancing transparency and credibility (Berger 2015).

### 3.2 Theoretical Framework: Cultural Safety

Cultural safety, first developed in New Zealand (Ramsden 2002), focuses on care that acknowledges power dynamics and respects the cultural identity of those receiving it. Rather than being defined by providers or institutions, safety in care is determined by the recipient (Nursing Council of New Zealand 2011). Nurses are encouraged to reflect on their own roles and consider how broader systems shape their relationships with patients (Curtis et al. 2019). In this study, this perspective guided both data collection and analysis, highlighting how trust, respect, and identity were expressed in participants' interactions with healthcare professionals.

### 3.3 Study Design

A focused ethnographic approach was employed (Cruz and Higginbottom 2013) to examine health‐related experiences and cultural meanings within a specific community. It allowed intensive engagement with participants in their natural settings while focusing on cultural norms, beliefs, and practices.

### 3.4 Setting and Participants

The study was conducted among 20 Laotian refugees (11 women, 9 men; aged 30s–70s), including 10 first‐generation, four 1.5‐generation, and six second‐generation. Eligible participants were adults of Lao ethnicity who had arrived in Japan as refugees under the Indochinese refugee resettlement program or were descendants of such refugees, and who had personal or family experiences with healthcare services in Japan. Participants were required to be able to communicate in either Japanese or Lao. In this study, first‐generation refugees were defined as individuals who had migrated to Japan as adults. 1.5‐generation refugees were defined as individuals who were born in Laos and migrated to Japan during childhood or early adolescence; they have early‐life experiences in Laos but grew up and received their education primarily in Japan. Second‐generation refugees were defined as those born in Japan to refugee parents. At the time of the study, participants had lived in Japan for ~30–40 years and were enrolled in Japan's public health insurance system. Recruitment was conducted at Buddhist temples that primarily serve Lao refugees and function as religious, cultural, and social hubs within the Lao community. These temples are predominantly used by people of Lao ethnicity and are not typically frequented by other ethnic groups (e.g., Hmong). While the temples serve mainly Lao refugees, Japanese supporters and researchers occasionally visit for support or collaborative activities. Recruitment occurred through temple networks serving as community hubs. Written informed consent was obtained in the participants' preferred language.

Fourteen participants were interviewed between September 2022 and March 2024, and six were added in August 2025 to strengthen information power (Malterud et al. 2016). Sampling maximized variation by generation, gender, and citizenship status, and included negative cases to deepen analysis. Table 1 outlines participant characteristics.

**TABLE 1:** Interviewee characteristics.

ID	Generation	Gender	Age (years)	Interview language	Citizenship	Birth country	Occupation	Approximate years living in Japan	Note summary
A	Second	Male	30s	Japanese	Stateless	Japan	Part‐time job	30 years	Expressed concern about nationality and equal treatment in healthcare
B	Second	Male	30s	Japanese	Stateless	Japan	Unemployed	30 years	Desired recognition beyond refugee status; wants to contribute to society
C	1.5	Male	40s	Japanese	Stateless	Laos	Part‐time job	30 years	Spoke about naturalization challenges and identity issues
D	Second	Female	30s	Japanese	Stateless	Japan	Office worker	30 years	Negotiates Lao caregiving customs and Japanese hospital practices
E	Second	Female	30s	Japanese	Stateless	Japan	Part‐time job	30 years	Blended home remedies with Japanese medicine and valued passing on traditions
F	1.5	Male	40s	Japanese	Japan	Laos	Part‐time job	35 years	Grateful to Japan; emphasized contribution to society
G	First	Male	60s	Lao (with interpreter)	Japan	Laos	Factory worker	35 years	Wished to spend final years in Japan; emphasized end‐of‐life (EoL) dignity
H	First	Female	70s	Lao (with interpreter)	Stateless	Laos	Part‐time job	40 years	Valued monks' presence during illness; acknowledged communication barriers in hospitals
I	First	Male	60s	Lao (with interpreter)	Stateless	Laos	Factory worker	35 years	Expressed refugee identity persists; wished for equality
J	First	Female	60s	Lao (with interpreter)	Japan	Laos	Part‐time job	40 years	Highlighted temple as a sanctuary; expressed wishes for EoL care
K	First	Female	60s	Lao (with interpreter)	Stateless	Laos	Part‐time job	40 years	Compared Lao and Japanese hospital care; wished to remain in Japan until EoL
L	First	Male	70s	Lao (with interpreter)	Japan	Laos	Unemployed	40 years	Desired home‐based EoL care; valued Lao customs related to death
M	First	Male	70s	Lao (with interpreter)	Japan	Laos	Unemployed	40 years	Discussed funerals blending Lao and Japanese traditions
N	First	Female	70s	Lao (with interpreter)	Japan	Laos	Unemployed	40 years	Expressed need for holistic care; emphasized traditional medicine
O	Second	Female	30s	Japanese	Stateless	Japan	Unemployed	30 years	Chose Japanese childbirth care; celebrated birth of child in line with Lao customs
P	1.5	Female	40s	Japanese	Japan	Laos	Unemployed	35 years	Faced infertility; combined religious and medical support
Q	Second	Female	30s	Japanese	Japan	Japan	Office worker	35 years	Negotiated bicultural identity around childbirth practices
R	1.5	Female	40s	Japanese	Japan	Laos	Unemployed	40 years	Naturalized in Japan; reflected on identity and citizenship
S	First	Female	70s	Lao (with interpreter)	Stateless	Laos	Temple assistant	40 years	Considered temple as essential for mental stability during COVID‐19
T	First	Male	70s	Lao (with interpreter)	Stateless	Laos	Unemployed	35 years	Reported loneliness when family was not allowed overnight in hospital

### 3.5 Data Collection

The first author, a Japanese nursing researcher, conducted all the interviews and participant observations. With participants' consent, she collected field notes, photographs, and supplementary materials during fieldwork. Audio‐recorded interviews were translated from Lao into Japanese, and study materials were translated from Japanese into Lao by a professional translation service to ensure linguistic accuracy. Interviews were guided by a semi‐structured interview guide, and all sessions were digitally audio‐recorded with participants' consent.

#### 3.5.1 Interviews

Twenty interviews were conducted: 4 in participants' homes and 16 at the community temple. Interviews began with open‐ended questions about life as a refugee, followed by focused questions on health, illness, and experiences with healthcare providers. Sample questions included “What is important to you in your daily life,” “How do you understand health and illness,” “What experiences have you had with healthcare providers in Japan,” and “What cultural practices are important for you in times of illness?”

Interviews lasted an average of 62 min; 10 were conducted in Japanese, while 10 were conducted in Lao with an interpreter fluent in both Lao and Japanese and experienced in refugee issues.

#### 3.5.2 Observations

Participant observation was conducted across 20 sessions (114 h) in temples, in participants' homes, and at community events such as Buddhist ceremonies and funerals. Field notes, photographs, and audio recordings were collected with participants' consent. The first author participated in religious and social activities and occasionally answered questions about Japanese healthcare, enriching contextual understanding.

### 3.6 Research Team

The research team comprised nursing scholars with expertise in refugee health, disaster nursing, clinical nursing, and chronic illness care. The first author received formal training in qualitative research methods and has ~10 years of experience in refugee support. The first author was responsible for study design, participant recruitment, data collection, transcription, and initial coding. Co‐authors contributed to peer debriefing, methodological consultation, and critical review of analytic decisions. All authors participated in the interpretation of findings and manuscript development.

### 3.7 Data Analysis

Data analysis followed an iterative process. Braun and Clarke's (2006) six‐phase thematic analysis was used as a flexible analytic method to code interview transcripts and identify patterns across the data. Focused ethnography informed the analytic process by guiding attention to cultural context, social practices, and generational positioning, and by integrating insights from participant observation and field notes into the interpretation of themes (Cruz and Higginbottom 2013). Interview transcripts and field notes were read repeatedly to achieve immersion. Lao interviews were translated into Japanese, checked through back‐translation, and verified with participants. Transcripts, field notes, and translations were securely stored on password‐protected devices, and identifying information was removed before analysis to ensure confidentiality.

Coding was conducted manually and without data analysis software. Codes were generated inductively from participants' accounts of health, illness, cultural values, and healthcare experiences and compared across interviews and observations. Through constant comparison, codes were clustered into categories and refined into broader themes. Cultural safety (Curtis et al. 2019; Ramsden 2002) served as an interpretive lens to examine issues of power, respect, and cultural identity in healthcare interactions.

Rigor was ensured in accordance with the criteria for rigorous qualitative research (Thorne 2016), including credibility, dependability and confirmability, and transferability. Credibility was enhanced through prolonged engagement, triangulation of interviews and participant observation, member checking, and peer debriefing. Member checking was conducted with all participants (*n* = 20). Of them, five participants provided feedback on preliminary interpretations, which was integrated into the analysis. Peer debriefing was conducted through regular discussions among the research team members. Co‐authors with expertise in qualitative research and refugee health critically reviewed coding decisions, emerging themes, and analytic interpretations throughout the analytic process. These strategies supported reflexivity and strengthened the trustworthiness of the findings. Dependability and confirmability were ensured through the maintenance of an audit trail documenting analytic decisions, coding processes, and reflexive memos. Transferability was enhanced by providing detailed descriptions of the research context, participant characteristics, and cultural settings, enabling readers to assess the applicability of the findings to other contexts.

Data saturation was observed in the final wave of interviews, as no new codes emerged and existing themes were repeatedly confirmed. We used the Standards for Reporting Qualitative Research (SRQR) guidelines (O'Brien et al. 2014) to structure and draft this manuscript, and the SRQR reporting checklist (O'Brien et al. 2025) when editing. This completed checklist is provided in Supporting Information.

### 3.8 Ethical Considerations

This study was conducted in accordance with the principles of the Declaration of Helsinki and was approved by the Nagoya University Graduate School Bioethics Review Committee (IRB No. 2020‐0421). Written and verbal informed consent was obtained from all participants in their preferred language (Japanese or Lao). Their identities were anonymized by allocating letters to each participant.

## 4 Results

### 4.1 Identified Themes

In this study, five themes emerged that reflected how Laotian refugees in Japan experienced healthcare and nursing care (Table 2). These themes encompassed how respect, trust, and identity were negotiated in healthcare encounters and how spiritual, cultural, and social continuity shaped participants' understandings of health and illness, foregrounding resilience, and vulnerability within the refugee experience.

**TABLE 2:** Themes and subthemes of experiences of cultural safety in nursing care.

Themes	Subthemes	Generational characteristics
1. Having a place of spiritual sanctuary	A unified space for collective gathering Peaceful venue for spiritual practices Temple as spiritual and emotional support Monks as sources of reassurance Impact of COVID‐19 on temple access and wellbeing Collective belonging through rituals	First generation relied on temples for emotional and spiritual support; 1.5‐generation emphasized the temple as a place of mental stability during crises; second generation participated in rituals during transitional moments.
2. Culturally congruent care	Negotiating between Lao and Japanese practices Respect for the Japanese healthcare system Harmony with Lao culture Preserving cultural identity in care Bicultural practices in everyday health management	First generation described tensions between Lao caregiving and Japanese hospital norms; 1.5‐generation acted as cultural translators; second generation balanced safety in Japanese care with Lao rituals.
3. Hopes for holistic care within the Japanese healthcare system (body–mind–spirit)	Hope for care that unites body and mind Acceptance of illness as part of life Navigating Japanese healthcare system Access to advanced treatment Trust in excellence of Japanese care Desire for integration of traditional medicine Purification rituals and health‐related beliefs	First generation valued spiritual and traditional medicine; 1.5‐generation combined religious support with fertility treatment; second generation relied on family for translation and blended practices
4. Recognition and equality from Japanese society	Right to equitable medical care Recognition of existence and contribution Overcoming language and institutional barriers Integration into Japanese society Identity beyond refugee labels Desire for recognition beyond refugee status	First generation expressed anxiety over legal status and refugee labeling; 1.5‐generation discussed naturalization and identity negotiation; second generation sought recognition beyond refugee status
5. Receiving culturally respectful end‐of‐life care	Assurance of dignified retirement Acceptance of illness and death Blended Lao–Japanese funeral practices Role of priests and rituals at the temple Wishes for dying at home with cultural customs	First generation emphasized dying in Japan with Lao rituals; 1.5‐generation supported temple‐based funerals; second generation coordinated bicultural end‐of‐life practices

#### 4.1.1 Theme 1: Having a Place of Spiritual Sanctuary

This theme was experienced differently across generations: While first‐generation participants relied on temples for emotional and spiritual support, younger generations engaged in rituals to maintain cultural continuity. Participants consistently emphasized the importance of having a spiritual sanctuary where they could spend time with peers and family, express themselves freely, and find peace. For many, this sanctuary was the temple—the community's religious and social hub—which functioned as a source of emotional comfort and cultural continuity, particularly during illness or hospitalization.

Getting sick is a sad thing, but I accept it. When I or my family fall sick, I need a place where I can feel tranquility. I get a little nervous in Japanese hospitals. It would be nice to have a place where I can feel tranquility. (J, female, 60s, first generation)

Additional interviews reinforced this role of the temple: “During COVID, when I could not go to the temple, I felt mentally unstable. The temple is truly my place of comfort.” (S, female, 70s, first generation).

Field notes also captured the affective weight of gathering at the temple. When ceremonies resumed, participants wore traditional Lao clothing and expressed their emotions. One refugee said tearfully, “We have become one despite being in a distant country. It took a long time to achieve this. There have been many hardships. But it is heartwarming to be able to gather here with the people of Laos.” Others around her nodded and wept (Field note, Temple, April 2023).

For younger generations, the temple also served as a place to transmit Lao traditions to their children. Many second‐generation participants attended Buddhist festivals with their families, emphasizing the importance of passing cultural values to the next generation. “I take my children to the temple for New Year and other ceremonies so that they can learn our culture and respect our ancestors.” (E, female, 30s, second generation).

Taken together, these accounts suggest that the temple was experienced as both a religious site and a cultural anchor, sustaining continuity and solidarity during times of illness and uncertainty.

#### 4.1.2 Theme 2: Culturally Congruent Care

Participants described the tensions of navigating Japanese healthcare while maintaining Lao cultural values. Many spoke of negotiating between practices at home and expectations in hospitals:

We had to align the rules of our country with Japan's. Hospitals and medical care in Japan are different from hospitals and medical care in my country. In my country, it is normal for family members to care for their family in the hospital, but in Japan, they leave it to the nurses. (K, female, 60s, first generation)

At home, we follow Lao culture, and outside the home, we follow Japanese culture. When our parents are sick, we care for them according to Lao culture in the home. In Lao culture, we respect our elders, provide care to the best of our ability, and sometimes pray for recovery. (D, female, 30s, second generation)

Field notes highlighted how cultural blending occurred in everyday life. For instance, one participant's home contained Japanese hospital medicines alongside Thai vitamins and Lao herbal remedies (Field note, K's Home, November 2023).

A second‐generation participant discussed childbirth practices:

When I became pregnant and gave birth, I had heard about traditional customs in Laos from my mother, but I wanted to give birth safely in Japan. My mother did not pressure me to follow any particular customs. My parents speak Japanese to their grandchild as much as possible. We celebrated the birth of our child in the Laotian style. (O, female, 30s, second generation)

These accounts show that participants continually balanced Lao caregiving norms and Japanese institutional expectations, underscoring the importance of healthcare that validates bicultural practices.

#### 4.1.3 Theme 3: Hopes for Holistic Care Within the Japanese Healthcare System (Body–Mind–Spirit)

Participants valued the advanced nature of Japanese medicine but also wished for recognition of traditional and spiritual approaches: “The mind and body are connected. I want to receive care that respects the mind and body” (N, female, 70s, first generation); “We understand that it is difficult to receive traditional medical care in Japan. We hope that it will be possible to practice traditional medicine based on our beliefs in some way” (I, male, 60s, first generation).

A younger 1.5‐generation participant spoke of combining religion and fertility treatment:

My husband and I wanted to have children, but we were unable to conceive. We consulted with a priest. We sought advice on cutting‐edge fertility treatment in Japan, but it was expensive, and we did not fully understand the system, so we were unable to make full use of it. (P, female, 40s, 1.5‐generation)

Field notes described a purification ritual in which participants prayed for good spirits to enter and evil spirits to leave (Temple, March 2023). In daily conversations, participants exchanged information on supplements, infertility treatment, and insurance, often relying on their children for translation. These narratives suggest that participants hoped for care that addressed the interconnectedness of body, mind, and spirit, integrating biomedical and traditional practices.

#### 4.1.4 Theme 4: Recognition and Equality From Japanese Society

Recognition and equality were interpreted through generational lenses: first‐generation participants voiced concerns about legal status, while 1.5‐ and second‐generation individuals reflected on identity negotiation and societal inclusion. Concerns about nationality and social identity were central to participants' healthcare experiences. Many feared discrimination or unequal treatment:

I have lived in Japan for a long time, but I do not have Japanese nationality. I can speak Japanese, and I have no problems visiting hospitals, but I have worried that I might not be treated fairly because I do not have Japanese nationality. (C, male, 40s, 1.5‐generation)

Identity and naturalization were ambivalent topics:

I was born in Laos and raised in Japan, but I was sometimes treated as a “foreigner” at hospitals. It was difficult because I had to apply for various things. Now, I have naturalized as a Japanese citizen. I chose a Japanese name using characters that I like. (R, female, 40s, 1.5‐generation)

Elders expressed complex feelings about lingering labels, such as “My refugee life has ended. However, Japanese society perceives us as refugees” (I, male, 60s, first generation). Simultaneously, others expressed gratitude and a desire to contribute: “I desire to be viewed as a person, not as a refugee. I am grateful to Japan, and I want to contribute to society” (F, male, 40s, 1.5‐generation). These accounts highlight that cultural safety requires not only clinical kindness but also recognition and equality beyond the “refugee” label.

#### 4.1.5 Theme 5: Receiving Culturally Respectful End‐of‐Life Care

As first‐ and 1.5‐generation refugees aged, concerns about end‐of‐life care and funeral practices became salient: “We wish to live true to ourselves until the end” (J, female, 60s, first generation); “I cannot go back to my home country to live, so I want to live in Japan until the end” (K, female, 60s, first generation).

Field notes described funerals that blended Japanese and Lao traditions, including donations for bereaved families and ceremonies held in both Japan and Laos (Field note, Temple, April 2024). Participants stated, “The priests from my home country stay at the temple so that we can have a funeral in our home country's style. This is very important for us, especially for the first generation” (N, female, 70s, first generation); “I am not worried about dying. I want to spend my final days at home, not in a hospital. When I die, I want to follow the cultural customs of my home country” (L, male, 70s, first generation). Participants underscored that end‐of‐life care must honor Lao cultural values while being situated in Japan, making cultural respect essential for dignity in dying.

## 5 Discussion

The five identified themes demonstrate that cultural safety was not an abstract principle but a practice embedded in the everyday realities of illness, healing, and family life. Importantly, this study extends the literature by showing how the cultural safety framework is interpreted in a refugee context in Japan, highlighting temples as community health resources, bicultural negotiations in care, and status‐related anxieties that shape perceived safety.

Interpretation and experience of each theme differed by generation. First‐generation participants emphasized spiritual and cultural continuity through temples, traditional caregiving, and Lao funeral practices. Meanwhile, 1.5‐generation participants often served as cultural and linguistic intermediaries, navigating fertility treatment, naturalization, and institutional systems. Finally, second‐generation participants expressed bicultural identities, integrating Japanese medical practices with selective preservation of Lao rituals, especially during childbirth and family caregiving. These generational perspectives enrich understanding of cultural safety as a lived and negotiated practice across life stages and healthcare encounters.

### 5.1 Cultural Safety as Lived Practice

Consistent with prior scholarship, cultural safety was described in relational and experiential terms—care that respected identity, reduced power imbalances, and was defined as safe by the care recipient (Curtis et al. 2019; Ramsden 2002). For participants, safety was achieved through not only clinical competence but also recognition: Being listened to, being treated with dignity, and having space for cultural and spiritual practices. Findings support existing work on refugee and immigrant health that highlights belonging and trust as being essential to care engagement (Edge and Newbold 2013; Grigg‐Saito et al. 2010). The study's contribution lies in showing how these dynamics unfold in Japan, where institutional support for refugees remains limited, and how cultural safety can correct systemic inequities.

### 5.2 Temples as Health Resources

A distinctive study contribution is identifying the temple as a cultural health infrastructure. For first‐generation participants, temples anchored collective belonging, offered emotional comfort during illness, and supported grief and funeral practices. These insights align with the prior qualitative finding that faith substantially influences refugees' health meanings and coping strategies, underscoring its importance as a health‐enabling resource (Bridi et al. 2023). For younger participants, temples provided continuity of identity during life transitions such as childbirth or infertility struggles. This extends previous community‐based refugee health research by showing that spiritual and religious sites can serve as culturally safe resources that support resilience and enable navigation of biomedical care (Grigg‐Saito et al. 2010).

### 5.3 Negotiating Bicultural Practices

Participants described continuous negotiation between Lao caregiving norms and Japanese institutional routines, evident in family presence at hospitals, childbirth practices, and the blending of Lao herbal remedies with Japanese pharmaceuticals. Second‐generation participants selectively preserved cultural rituals while adopting Japanese medical standards, whereas 1.5‐generation participants often served as linguistic and cultural intermediaries within families and hospitals. These findings reflect Leininger's (2006) theory of culture care diversity and universality by illustrating both culturally specific expressions of care shaped by migration histories and generational positioning (diversity), and core caring values that remain meaningful across generations (universality). However, this study's analysis emphasizes participants' own meanings rather than prescriptive cultural knowledge. For nursing, the implication is not to provide “cultural facts” but to engage in situated negotiation: Asking what matters to each patient and family and adapting practices accordingly.

### 5.4 Holistic Approaches to Care

Participants valued Japan's advanced medical system but wished for acknowledgment of the interconnectedness of body, mind, and spirit. Their accounts of seeking fertility treatment alongside religious rituals highlight the importance of holistic approaches that integrate biomedical and traditional understandings. International research indicates that culturally attuned whole‐person care fosters trust and adherence in refugee populations (Higginbottom et al. 2014; Magwood et al. 2022).

### 5.5 Recognition and Equality

Concerns about nationality, legal status, and enduring refugee labels were deeply tied to healthcare experiences. Some participants expressed gratitude toward Japanese healthcare, while others carried complex feelings about being perceived primarily as “refugees,” even after decades of residence. These narratives reflect the intersection of structural barriers—such as insurance rules and naturalization procedures—with personal identity and belonging (Khin et al. 2025; Morita et al. 2021).

### 5.6 End‐of‐Life Care and Dignity

Aging participants emphasized their desire to remain in Japan while honoring Lao end‐of‐life traditions. Funerals blending Lao and Japanese customs exemplified how cultural continuity is central to dignity at the end of life. This resonates with international research that identifies cultural meanings as fundamental to palliative care (Schill and Caxaj 2019).

### 5.7 Toward Equity‐Oriented Nursing Practice

Findings align with the broader framework of equity‐oriented care, which integrates cultural safety, trauma‐ and violence‐informed approaches, and contextual tailoring (Browne et al. 2016). The study extends this literature by (a) demonstrating how community temples function as health‐enabling infrastructures; (b) highlighting status‐related anxieties as mechanisms shaping refugees' sense of safety; and (c) detailing how bicultural negotiations around caregiving, childbirth, and end‐of‐life rituals form concrete sites where cultural safety is enacted. Thus, it highlights the value of reflective nursing practice that moves beyond cultural sensitivity toward relational and equity‐oriented care.

### 5.8 Strengths and Limitations

This study has several strengths. By adopting a focused ethnographic design and recruiting participants through Buddhist temples established by Lao refugees, the study captured culturally embedded meanings and practices related to healthcare experiences that may not be visible in clinical settings. The inclusion of first‐, 1.5‐, and second‐generation participants also enabled exploration of both generational differences and continuities in perceptions of culturally safe nursing care.

However, several limitations should be acknowledged. Participants were recruited primarily through temple‐based networks, which may have resulted in recruitment bias toward individuals who are socially connected, culturally engaged, and actively involved in community and religious activities. Temple‐engaged members may have stronger social support networks, greater access to culturally shared information, and more opportunities for collective meaning‐making around health and care. As a result, the experiences of Lao refugees who are less connected to temples, more socially isolated, or who primarily navigate healthcare outside community‐based networks may not be fully represented. Therefore, the findings may not be generalizable to all refugee populations in Japan. Nonetheless, they offer contextually grounded insights into how cultural safety is experienced within a long‐settled Lao refugee community. Future research should include refugees with diverse levels of community engagement to further examine variations in culturally safe nursing care experiences.

## 6 Conclusion

This study clarified what constitutes cultural safety for Laotian refugees living in Japan. For participants, cultural safety was realized through everyday practices that affirmed identity, nurtured spiritual–social continuity, and addressed status‐related anxieties while engaging with advanced biomedical care. Specifically, it was grounded in access to a spiritual sanctuary; culturally congruent caregiving; holistic approaches that integrate body, mind, and spirit; recognition and equality in Japanese society; and culturally respectful end‐of‐life care.

This study's results resonate with established frameworks such as equity‐oriented care (Browne et al. 2016) but extend this scholarship by contributing insights unique to refugee experiences in Japan. Temples were conceptualized not only as religious venues but also as health resources. Uncertainties around nationality and residence status emerged as psychological burdens shaping care experiences. Finally, bicultural practices were negotiated across generations in concrete contexts such as childbirth, infertility, and family caregiving.

These findings suggest that cultural safety is not an abstract ideal but an enacted reality, co‐constructed in relationships between refugees, families, communities, and nurses. By engaging with refugees' lived meanings of health and illness, nursing practice can move beyond cultural sensitivity toward relational, contextually tailored, and equity‐oriented care.

## 7 Relevance for Clinical Practice

This study has several implications for clinical nursing practice. Given the role of community temples as trusted cultural and spiritual resources for Lao refugees, nurses may consider partnering with temple‐based communities as health assets. Collaborative engagement with temples may support culturally appropriate referral pathways for spiritual support, bereavement care, and health education, particularly for refugees who experience barriers to accessing formal healthcare services (Grigg‐Saito et al. 2010).

The findings also highlight the importance of routinely engaging in “what matters” conversations with refugee patients. By eliciting valued practices such as family presence, prayer, and the use of traditional remedies, nurses can negotiate feasible adaptations within Japanese clinical norms while maintaining patient safety (Leininger 2006). Attending to the integration of body, mind, and spirit may further enhance culturally safe care: For example, by ensuring access to quiet spaces for prayer, discussing traditional remedies without judgment, and addressing potential interactions with biomedical treatments (Higginbottom et al. 2014).

In addition, nurses play a key role in recognizing and reducing status‐related anxieties that may undermine refugees' sense of safety in healthcare settings. Proactively explaining health insurance systems, consent processes, and institutional rules, checking understanding through teach‐back, and facilitating access to professional interpreters may help mitigate these anxieties (Khin et al. 2025; Morita et al. 2021).

Finally, culturally respectful end‐of‐life care requires early and ongoing communication with patients, families, and, where appropriate, temple leaders. Discussing ritual preferences in advance and coordinating care across clinical and community settings may support more equitable and culturally safe end‐of‐life experiences (Schill and Caxaj 2019). At the team level, ongoing reflective practice focused on power, positionality, and bias—grounded in principles of cultural safety rather than static cultural knowledge—can further strengthen equity‐oriented nursing practice (Curtis et al. 2019; Nursing Council of New Zealand 2011).

## Author Contributions

**Maki Nakajima:** data curation, formal analysis, funding acquisition, investigation, resources, writing – original draft, writing – review and editing; **Ikumi Honda:** formal analysis, validation, project administration, resources, supervision, writing – review and editing; **Tomoko Doi:** formal analysis, validation, writing – review and editing. All authors approved the final version of the manuscript to be published. All authors contributed to the study's conceptualization and methodology.

## Funding

This work was supported by the Japan Society for the Promotion of Science (24K20273).

## Ethics Statement

The procedures used in this study adhere to the tenets of the Declaration of Helsinki. The study protocol was reviewed and approved by the Nagoya University Graduate School Bioethics Review Committee (IRB No. 2020‐0421).

## Consent

Written informed consent to take part in the research and publish the findings was obtained from all study participants.

## Conflicts of Interest

The authors declare no conflicts of interest.

## Supporting information


**Data S1:** Supporting Information.

## Data Availability

The data that support the findings of this study are available on request from the corresponding author. The data are not publicly available due to privacy or ethical restrictions.
